# Characteristics of Menstrual Cycle in Shift Workers

**DOI:** 10.5539/gjhs.v5n3p163

**Published:** 2013-02-28

**Authors:** Mirsaeed Attarchi, Hamidreza Darkhi, Maryam Kashanian, Mahshad khodarahmian, Mandana Dolati, Mostafa Ghaffari, Elham Mirzamohammadi, Saber Mohammadi

**Affiliations:** 1 Occupational Medicine Department, School of Medicine, Tehran University of Medical Sciences, Tehran, Iran; 2 Department of Obstetrics & Gynecology, School of Medicine, Tehran University of Medical Sciences, Tehran, Iran; 3 Tehran University of Medical Sciences, Tehran, Iran; 4 Department of Pathology, Legal Medicine Organization, Tehran, Iran; 5 Brain and Spinal Cord Injury Research Center (BASIR), Tehran University of Medical Sciences, Tehran, Iran

**Keywords:** menstruation disorder, shift work, occupational exposure, female worker, reproductive health

## Abstract

**Background:**

In this study, the characteristics of menstrual cycle in shift workers employed in the pharmaceutical industry are investigated.

**Method:**

This study was conducted in a pharmaceutical industrial complex in Tehran in 2012. 406 female workers in packaging units were studied on the menstrual cycle characteristics. The studied workers were divided into two groups of shift workers and non-shift workers and were compared in terms of the frequency of menstrual disorder (short-term cycle, long-term cycle, irregular cycle and bleeding during menstrual cycle) as well as hormonal values (FSH, LH, TSH, and Prolactin).

**Results:**

The odds ratio (OR) for menstrual disorder in the shift workers was 5.54 (95% CI=2.78-11.02) compared to the non-shift workers. The mean difference of hormonal values (except prolactin) between shift workers and non-shift workers was not significant (P> 0.05).

**Conclusion:**

This study suggests that shift work may disrupt the menstrual cycle.

## 1. Introduction

Given the increasing speed of industrialization process of countries and also in order to increase the production and productivity in manufacturing sectors, shift work is inevitable and expanding. In the United States, in the last two decades, about 27 percent of men and 16 percent of women have experienced shift work (Pati et al., 2001). The results of previous studies indicate that about 15 million people in the United States are shift workers (Lawson et al., 2011). According to the statistics of International Labor Organization (ILO), approximately 15 to 30% of workforce in developing countries is shift workers (Dumont, 1985). On the other hand, in recent years, the number of women as workforce is increasing. Also, a lot of these workers are in the reproductive ages and it is necessary to pay attention to the effects of occupational exposures on the reproductive system (Zenz et al., 1994).

Some studies were conducted in previous years on the health effects of shift work. In previous studies, a positive significant relationship was found between shift work and sleep problems, digestive problems and adverse effects on the cardiovascular system, but the impact of shift work on the reproductive system has not been adequately addressed. The effect mechanism of working shift on the reproductive system is not completely clear, but shift workers are usually suffering from sleep disorder and increased disorder risk in the physiological function including hormonal disturbances and disruption in the circadian rhythm, which are considered as possible reasons of the relationship between shift work and abnormal menstrual cycle (Navara & Nelson, 2007). The duration and pattern of menstrual cycle are considered as women’s reproductive health indicators (Harlow & Campbell, 2008; Lisabeth et al., 2004). Irregular menstrual cycle patterns, including short or long-term cycles are associated with reduced levels of women’s productivity (Axmon et al., 2006; Rowland et al., 2002; Small et al, 2006). Menstrual disorder is considered a relatively common disorder in developed and developing countries (Siobán, 2000). Menstrual dysfunction reduces the quality of life, increases absenteeism, and imposes significant costs to the industry. It is estimated that in the United States, the overall cost of lost working days due to menstrual dysfunction is 94 to 308 million dollars for each lost day (US Bureau of the Census, 1994). Previous studies have examined the relationship between shift work and the duration and pattern of menstrual cycle, but most of these studies had small sample sizes or short duration of shift working (Chung et al., 2005; Hatch et al., 1999; Labyak et al., 2002; Lohstroh et al., 2003; Messing et al., 1992; Su et al., 2008). The results of a study by Miyauchi et al. (1992) indicated that night working and shift working can lead to irregular menstrual cycles possibly by disrupting the circadian system and the reproductive hormone. Also, in a study conducted on workers in a textile factory in China, Lohstroh et al. (2003) indicated that shift working can cause irregularity in the duration of menstrual cycle. On the other hand, the pharmaceutical industry is an important industry in Iran and a large workforce are employed in this industry. In 2004, it was reported that 65 pharmaceutical companies, 20 of which are too large, are operating in Iran (Cheraghali, 2006). In this study, the characteristics of menstrual cycle in female shift workers who are working in the pharmaceutical industry are investigated.

## 2. Methods

### 2.1 Study Design and Population

This study was conducted in a pharmaceutical company located in the outskirt of Tehran in 2012. The researchers examined the characteristics of menstrual cycle in female workers employed in this industry. All women working in the packaging units of factory who were in the reproductive age (before menopause) and had at least one year of work experience were included in the study. All the necessary information, including demographic, medical and employment data were collected through direct interviews and from medical records in a questionnaire. The questionnaire contained questions about age, work experience, education level, marital status, height, weight, smoking habit, regular exercise, tea consumption, carrying heavy loads, long standing at work, assessment of the individuals’ job satisfaction and job stress, shift work status, use of medication, age at the first menstruation, pregnancy and menstrual cycle characteristics (including cycle duration, duration and amount of bleeding, bleeding during the cycle, regular or irregular menstrual cycles and dysmenorrhea) and previous job or a second job.

The exclusion criteria included pregnancy, history of hysterectomy, ovariectomy, diseases like Asherman’s syndrome and also history of endocrine disorders (progesterone deficiency, polycystic ovary syndrome, thyroid disorders and diabetes mellitus), continuous and non-occupational exposure to any organic solvents and pesticides. Also, those who were shift workers in previous jobs or second jobs as well as those who had changes in their working shift (from shift working to, day working or vice versa) were excluded from the study.

To investigate the occupational exposures effective on the reproduction system, environmental measurements were conducted on organic solvents and other chemicals in the packaging units under study. The results indicated no or a negligible amount of chemicals in the workplace. In the factory packaging units, 467 workers were employed. The under study workers were divided into two groups of shift work and none-shift work. After considering the inclusion and exclusion criteria, 113 people were placed in shift work group and 293 people were included in none-shift work group.

### 2.2 Ethical Approval

Before entering the study, all participants signed a written consent form. Also, they could exit the study at any time they wanted. This study was approved by the Ethics Committee of Tehran University of Medical Sciences.

### 2.3 Assessment of Menstrual Patterns and Covariance

In this study, the menstrual cycles of subjects were evaluated during the past year using the criteria including mean duration of menstruation, changes in duration of different cycles, bleeding time, amount of bleeding and the presence of dysmenorrhea. Menstrual disorder was defined as the existence of one of the four following characteristics in the menstrual cycle: short cycles, prolonged cycles, irregular cycles and bleeding or spotting between cycles (inter menstrual bleeding) (Ronda et al., 2009). The short-term cycle was defined as a menstrual cycle shorter than 21 days. Also, the prolonged cycle was defined as a menstrual cycle longer than 35 days (Cho et al., 2001).

In this study, the menstrual cycle pattern was obtained based on the question “What is the average duration of your menstrual cycle?” In other words, “How much does it take from the first day of your menstruation in a cycle to the first day of the menstruation in the next cycle?”

The onset of menstrual cycle was defined as the first day of two consecutive days with the onset of bleeding that at least in one of them the bleeding severity is more than spotting (Jukic et al., 2008). Besides, the cycle irregularity was defined as having more than 7 days for changes in the menstrual cycle duration constantly or in most cases. Likewise, the regular cycle was defined as having a maximum of 7 days for changes in the duration of cycles.

To determine the changes in menstrual cycles duration, participants were asked “whether the number of days from the beginning of a cycle to the next cycle are identical or not? And, if different, how many days is the difference?” (Lawson et al., 2011).

To measure the bleeding duration, participants were asked “on average, how many days does it take your menstrual bleeding”. To measure the amount of bleeding, participants were asked “on average, how many tampons do you use during each day of your menstrual cycle?”

In this study, Menstrual cycle discomfort was defined as low back pain or abdominal discomfort in bleeding time and dysmenorrhea was defined as at least 2 days of menstrual cycle discomfort during menstrual bleeding (Chen et al., 2000).

The day working was defined as work in a day with a constant pattern and the shift working was defined as working in all types of shifts including fixed evening shifts and rotating shifts except the fixed day time shift (day working) (Zhu et al., 2004).

Job satisfaction and job stress - which were determined through self-perception in this study - were classified in three groups of low, medium and high for job satisfaction and four groups of no, low, medium and high for job stress (Chung et al., 2005).

In this study, the regular exercise was defined as doing at least 30 minutes of exercise a day for at least 3 times a week or more (Lee et al., 2009).

To measure the amount of hormones of Follicle Stimulating Hormone (FSH), Luteinizing Hormone (LH), Thyroid Stimulating Hormone (TSH), Prolactin (PRL), estrogen and progesterone, the blood samples of subjects were measured on the third day of menstruation.

### 2.4 Statistical Analysis

The mean, standard deviation, and range of variables were calculated. The t-test and chi-squared test were used to compare different variables. Also, the logistic regression analysis was used to adjust the confounding factors and to more accurately assess the relationship between exposure to shift working and menstrual disorders. In this study, menstrual disorder was considered dependent variable and age at menarche, regular exercise, marital status, age, body mass index (BMI), work experience, self-perceived job satisfaction and self-perceived job stress were considered independent variables. The significance level was 0.05. The outputs of statistical analyses were expressed based on the odds ratio (OR) with 95% of confidence interval. The SPSS-11 software was used for statistical analyses.

## 3. Results

In this study, 406 female workers in the packaging units of the pharmaceutical factory were investigated. 293 people (72.2%) were day workers and 113 people (27.8%) were shift workers. The average age of all 406 workers was 31.3 years, with a range of 22-43 years. The average work experience for all workers was 8.7 years, with a range of 1-23 years. The mean BMI for all workers was 23.6 kg/m^2^, with a range of 18-35 kg/m^2^. The clinical, demographic and occupational variables among shift workers and day workers are compared in Table 1. As shown in Table 1, there was not a significant different between the two groups in terms of demographic, clinical and occupational factors (p> 0.05), except for the frequency of past history of pregnancy that was significantly higher in the day work group compared with that of the shift work group (p <0.05).

**Table 1:** Demographic characteristics and effective variables of menstrual cycle of all 406 under study workers

Variable	Day time workers (N=293)	Shift workers (N=113)	P-value
Age (year) Mean (SD)	31.3 (4.9)	31.5 (5.3)	0.720
Work of duration (year) Mean (SD)	8.9(4.9)	8.3 (4.9)	0.307
Body mass index (kg/m^2^) Mean (SD)	23.9 (3.4)	23.3 (3.2)	0.096
Age of menarche (year) Mean (SD)	12.6 (1.3)	12.7 (1.2)	0.359
Marital status N (%)			
Single	40 (13.6)	14 (12.4)	0.166
Married	246 (83.9)	92 (81.5)	
Divorced/Separated	7 (6.1)	7 (2.5)	
Pregnancy history Yes (%)	211 (72.0)	65 (57.5)	0.034
Use of OCP Yes (%)	45 (15.3)	17 (15.0)	0.756
Educational levels N (%)			
Guidance and high school a	115 (39.3)	47 (41.6)	0.183
High school graduate b	166 (56.6)	61 (53.9)	
Associate degree c	12 (4.1)	5(4.5)	
Tea consumption Yes (%)	168 (57.3)	63 (55.7)	0.731
Regular exercise Yes (%)	44 (15.0)	20 (17.6)	0.300
Standing duration (>3 h) Yes (%)	61(20.8)	25 (22.1)	0.342
Heavy lifting (>10 kg) Yes (%)	15 (5.1)	6 (5.3)	0.561
Perceived job satisfaction N (%)			
Low	155 (52.9)	51 (45.2)	0.136
Medium	94 (32.0)	36 (31.8)	
High	44 (15.1)	26 (23.0)	
Perceived job stress N (%)			
None	20 (6.8)	6 (5.4)	
Low	96 (32.7)	33 (29.2)	0.496
Medium	126 (43.1)	58 (51.3)	
High	51 (17.4)	16 (14.1)	

a Between 8-12 years of education in school

b At least 12 years of education in school

c An educational level between High school graduate and Bachelor of Science (at least 2 years of education in university)

The mean duration of menstrual cycle for all 406 workers was 26.2 days, with a range of 16-40 days. The mean duration of bleeding time for all 406 workers was 4.9 days, with a range of 3-7 days and the mean number of used tampons per day was 3.6, with a range of 1-8. 205 workers (50.5%) were suffering from dysmenorrhea. 10.5% of workers had irregular cycles and 3.4% of workers had inter menstrual bleeding. The duration of menstrual cycles for 88% of workers was between 21 and 35 days. However, it was less than 21 days for 3.9% of workers, and more than 35 days for 8.1% of workers. Overall, 59 workers (14.5%) were suffering from menstrual disorders.

The average values of hormonal levels in both groups of day work and shift work are compared in Table 2. It was lower in the shift work group compared with the day work group. However, this difference was not significant (p> 0.05).

**Table 2:** Hormonal Characteristic of under study workers

Measurements		Day time workers (N=293)	Shift workers (N=113)	P-value
FSH (mlU/ml)	Mean (SD)	7.24 (1.51)	7.13 (0.44)	0.538
LH (mlU/ml)	Mean (SD)	5.00 (0.75)	4.85 (0.89)	0.072
TSH (mlU/ml)	Mean (SD)	2.98 (0.70)	2.89 (0.98)	0.356
PRL (ng/ml)	Mean (SD)	11.03 (2.74)	11.82 (3.07)	0.017

The menstrual cycle characteristics in both groups of day work and shift work are summarized in Table 3. As shown in the Table, the average length of menstrual cycle, the average of bleeding time and the number of tampons used in shift workers are significantly higher than that of day workers (p <0.05). Also, dysmenorrheal frequency, short-term menstrual cycle, long-term menstrual cycle, irregular menstruation and inter menstrual bleeding in shift workers are significantly higher than that of day workers (p <0.05). Finally, the prevalence of menstrual disorders in shift workers is significantly higher than that of day workers (P <0.001, OR = 3.8, 95% CI = 2.2-6.8).

**Table 3:** Menstrual cycle characteristics of under study workers

Variables	Day time workers (N=293)	Shift workers (N=113)	P-value
Length of cycle (day) (Mean± SD)	25.63±3.20	27.73±5.37	0.001
Duration of bleeding (day) (Mean± SD)	4.86±1.07	5.12±0.87	0.024
Amount of flow (number of used tampons) (Mean± SD)	3.47±1.28	3.97±1.71	0.001
Dysmenorrhea (yes) N (%)	130 (44.3)	75 (66.3)	0.001
Amount of flow (number of used tampons) 4≤	112 (38.2)	69 (61.0)	0.001
Short menstrual cycle (21 days>) yes N (%)	6 (2.0)	10 (8.8)	0.003
Long menstrual cycle (35 days<) yes N (%)	15 (5.1)	18 (15.9)	0.001
Irregular menstrual cycle Yes N (%)	21 (7.1)	22 (19.4)	0.031
Bleeding during menstrual cycle Yes N (%)	5 (1.7)	9(7.9)	0.014
Menstrual disorder Yes N (%)	27 (9.2)	32 (28.3)	0.001

Also, after adjusting the confounding factors, the logistic regression analysis indicated a significant association menstrual disorder and shift work (p <0.05). In addition, a significant relationship was found between menstrual disorder and work experience, age at menarche, regular exercise, high perceived job stress and low job satisfaction (P <0.05) (Table 4).

**Table 4:** Association between menstrual disorder and study variables using logistic regression analysis

Variable	Status	Adjusted OR	95% CI	P-value
Shift work	Day time workers (n=293)	1.00	-----------	------
	Shift workers (n=113)	5.54	2.78-11.02	0.001
Age (year)	≤31 (n=216)	1.00	-----------	-------
	>31 (n=190)	1.90	0.87-4.14	0.104
Age of menarche (year)	11-16 (n=384)	1.00	--------	--------
	> 16 or <11 (n=22)	3.01	1.33-6.12	0.023
Work of duration (year)	≤ 7.5 (n=201)	1.00	-----------	-------
	> 7.5 (n=205)	2.74	1.24-6.07	0.013
Body mass index (kg/m2)	≤ 23.3 (n=200)	1.00	-----------	-------
	> 23.3 (n=206)	1.48	0.77-2.87	0.236
Marital status	Single(n=54)	1.00	-----------	------
	Married (n=336)	1.62	0.30-8.72	0.571
	Divorced/Separated (n=14)	0.86	0.11-6.34	0.890
Regular exercise	Yes(n=64)	1.00	-----------	-------
	No(n=342)	9.10	2.28-36.26	0.002
Perceived job satisfaction	High (n=206)	1.00	-----------	-------
	Medium (n=130)	2.17	0.94-5.01	0.068
	Low (n=70)	4.88	2.20-10.81	0.001
Perceived job stress	None (n=26)	1.00	------	-------
	Low (n=129)	1.78	0.84-7.34	0.831
	Medium (n=184)	5.41	0.92-12.67	0.114
	High (n=67)	7.35	1.32-16.63	0.033

Moreover, the results of regression analysis indicated that in the shift workers group with a job experience of more than 7 years, odds ratio (OR) for menstrual disorder was higher than that of the day workers group with a job experience of 7 years or less and the non-shift work group (Table 5).

**Table 5:** Association^a^ between menstrual cycle characteristics and shift work duration

Shift work status (years)	Menstrual Cycle Characteristics OR (95%CI)				

	Dysmenorrhea	Irregular cycle	Short cycle (21 days>)	Long cycle (35 days<)	Menstrual disorder
Non-shiftworker^b^	1.00	1.00	1.00	1.00	1.00
≤7 years	3.38 (1.35-8.45)	3.74 (1.55-9.02)	2.68 (1.24-6.72)	2.81 (1.12-8.32)	2.96 (1.25-7.00)
>7 years	7.65 (2.77-21.08)	9.93 (4.42-22.30)	5.01 (1.83-13.41)	5.52 (2.01-15.00)	6.88 (3.12-15.17)

a Adjusting for age, Age of menarche, body mass index, Marital status, regular exercise, Perceived job satisfaction and Perceived job stress using logistic regression analysis

b Reference category

## 4. Discussion

The pattern of menstrual cycles in females is considered an important indicator of the reproductive health. On the other hand, changes in menstrual function can propose shift workers’ intolerance toward shift working (Labyak et al., 2002). The results of our study indicated that even after adjustment of confounding factors, there is a significant relationship between shift working and menstrual cycle disorder.

Chung et al. (2005) studied the relationship between menstrual cycle characteristics and lifestyle factors and occupational factors in nurses in Taiwan. The results indicated that there is a significant association between “occupational and lifestyle factors” and “dysmenorrhea and menstrual cycle function”. In that study, for 60% of fixed night working nurses with regular menstrual cycles, the duration of menstrual cycle was less than 25 days, and it was significantly higher than nurses in other working schedules (p <0.05). Lawson et al. (2011) studied the pattern of menstrual cycle in nurses with rotating shift work. The results indicated that 10% of nurses had irregular menstrual cycles. The duration of menstrual cycle for 70% of nurses was between 26 and 31 days, 16% between 21 and 25 days, 1% less than 21 days, 11% between 32 and 39 days and 1% more than 40 days. For nurses with a history of more than 20 months in rotating shift, the frequency of irregular menstrual cycles (adjusted RR = 1.23 [CI = 1.14 -1.33]) and also the frequency of menstrual cycles (adjusted RR = 1.27 [0.99 -1.62]) were less than 21 days or more than 40 days (adjusted RR = 1.49 [1.19 -1.87]) compared with the workers without rotating shifts. In the irregular menstrual cycle patterns and the menstrual cycle longer than 40 days, a relationship was observed between dose-response and the increase of months of rotating shift working. The results of our study also suggested that the frequency of all types of menstrual disorders including short-and long-term menstrual cycles, irregular menstrual cycle and dysmenorrhea are higher for shift workers with more than 7 years of job experience compared with that of shift workers with equal or less than 7 years of job experience and day workers. In general, the results of the study by Lawson et al. (2011) indicate that there is a moderate association between shift work and the menstruation pattern and duration of menstrual cycle. Overall, in this study, an increased risk was observed for long-term and short-term menstrual cycles and irregular menstrual cycles in nurses who work in rotating shifts.

Our study was not found a significant association between BMI and menstrual disorders using logistic regression analysis, but in the study of Lawson et al. (2011), a dose-response relationship was found between self-reported body mass index and menstrual cycle outcomes.

In the study of Labyak et al. (2002), 68 nurses under 40 years of age were studied in terms of menstrual cycle, pregnancy outcomes, and sleep problems. 53% of women experienced changes in their menstrual cycle when shift working. Also, shift working nurses were more subject to sleep disorder which may cause disturbances in the menstrual cycle regulation.

In a study on poultry slaughterhouse and cannery workers, it was found that shift work (with variable working schedule) was associated with long-term menstrual cycle (more than 33 days) (OR = 2.4, 95% CI = 1.0-5.5) and irregular menstrual cycle (OR = 2.0, 95% CI = 1.2-3.6) (Messing et al., 1992). Higher risk ratio in this study could be due to different definitions of long-term menstrual cycle and shift working.

In a study by Su et al. (2008) on workers of a factory in Taiwan that had 12-hour shift working and the day and night shifts rotated every 4 months, an increased risk was associated with long-term menstrual cycle (more than 35 days) or short-term menstrual cycle (less than 25 days) in shift workers.

The results of our study indicated increased prevalence of dysmenorrhea among shift workers compared with day workers. The results of a study by Yao et al. (2009) showed that shift working may increase the risk of premenstrual syndrome.

The menstrual cycle is identified through the reproductive hormone secretion with cyclical patterns. Although the impact mechanism of shift working on menstrual cycle is not entirely clear, but changes in the circadian rhythm through sleep disorder or changes in the melatonin production may affect the reproductive hormones adjustments which are responsible for controlling the menstrual cycle (Epstein, 1997). In humans, the relationship between time and intensity of light exposure and menstrual cycle parameters such as duration of menstrual cycle is not entirely clear (Barron, 2007). However, there are possible reasons and hypotheses for it. For example, previous studies have indicated a relationship between the urine levels of melatonin metabolites and work schedule and amenorrhea (Brzezinski et al., 1988; Hansen et al., 2006; Schernhammer et al., 2004; Schernhammer et al., 2006).

The results of our study suggest that hormonal levels including FSH, LH and TSH in shift workers were lower than day workers, but the difference was not significant. However, the mean prolactin level was significantly higher in shift workers than day workers.

During sleep, the secretion of pituitary LH is largely restricted (Baumgartner et al., 1993; Hall et al., 2005). Thus, changes in sleep/wake-up patterns in shift workers can lead to changes in LH secretion and therefore changes in the duration of menstrual cycle. The increase in prolactin secretion may be associated with changes in the duration of menstrual cycle, lack of ovulation and changes in the duration and amount of menstrual bleeding (Hatch et al., 1999).

In a study, Lohstroh et al. (2003) found that some of rotating shift workers had shorter menstrual cycles (27-31 days) and some had longer menstrual cycles (33-46 days). Hormonal profiles during menstrual cycles indicated that their changes during cycles are primarily caused by the differences during the follicular phase of the cycle, not by their changes during the luteal phase. The results of the study suggest an association between disorders in rotating shift work and longer menstrual cycle by delaying the ovulation day and prolongation of the follicular phase. Some workers with longer menstrual cycles (33-46 days) had irregular work schedule and shifts, but it was not true in those with shorter menstrual cycles (27-31 days). Also, the urinary level of FSH in the pre-LFPT phase of longer menstrual cycle decreased compared with the pre-LFPT (Luteal-Follicular Phase Transition) of shorter menstrual cycle. This result confirms that changes in the work schedule can cause irregularities in the menstrual cycle duration. In general, the results of this study indicate that LFPT is considered a sensitive and specific period in the menstrual cycle, when the environmental stressors can have adverse effects on the ovarian function.

In a study by Hatch et al. (1999), the prevalence of short-term menstrual cycle and the inadequate luteal phase in shift working nurses were higher than nurses who had fixed day or night shifts.

In our study, the frequency of menstrual disorder among workers with high work stress and low job satisfaction was higher than that of the control group. Psychological stress can activate the corticotropin release in the nervous system and may cause menstrual dysfunction (Chrousos et al., 1998). Environmental stress can interfere with various endocrine profiles, especially a reduction of estrogen and gonadotropin (Warren & Perlroth, 2001).

Among the limitations of our study is its cross-sectional characteristic, so there are constraints in determining the causal relationships. Also, the self-reported work schedule and the menstrual cycle characteristics may lead to misclassified participants. However, the information about the menstrual cycle characteristics only in the past year was collected, which may reduce the impact of misclassification. The participants were asked about the outcomes in the past, so recall bias is likely. Women reported their cycle characteristics without applying menstrual diaries. Obviously, the prospective data collection and using menstrual diaries can be helpful in increasing the quality of study.

It was better to used standard questionnaires to study job stress and job satisfaction. However, in this study, more questions could reduce the number of people who completed the questionnaire.

## 5. Conclusions

The results of our study indicated that the frequency of menstrual cycle disorder was higher in shift workers than that of the fixed day workers. To demonstrate the relationship between shift work and menstrual cycle disorder, further studies are recommended, especially of the prospective type. Also, in the periodic medical examinations, it is recommended to pay special attention to the pattern of menstrual cycle in shift workers.

## References

[ref1] Axmon A, Rylander L, Albin M, Hagmar L (2006). Factors affecting time to pregnancy. Hum Reprod.

[ref2] Barron M (2007). Light exposure, melatonin secretion, and menstrual cycle parameters: an integrative review. Biol Res Nurs.

[ref3] Baumgartner A, Dietzel M, Saletu B, Wolf R, Campos-Barros A, Gräf K. J, Mannsmann U (1993). Influence of partial sleep deprivation on the secretion of thyrotropin, thyroid hormones, growth hormones, prolactin, luteinizing hormone, follicle stimulating hormone, and estradiol in healthy young women. Psychiatry Res.

[ref4] Brzezinski A, Lynch H, Seibel M, Deng M, Nader T, Wurtman R (1988). The circadian rhythm of plasma melatonin during the normal menstrual cycle and in amenorrheic women. J Clin Endocrinol Metab.

[ref5] Chen C, Cho S. I, Damokosh A. I, Chen D, Li G, Wang X, Xu X (2000). Prospective study of exposure to environmental tobacco smoke and dysmenorrheal. Environ Health Perspect.

[ref6] Cheraghali A (2006). Iran pharmaceutical market (editorial). Iran J Pharm Res.

[ref7] Cho S. I, Damokosh A. I, Ryan L. M, Chen D, Hu Y. A, Smith T. J, Xu X (2001). Effects of exposure to organic solvents on menstrual cycle length. J Occup Environ Med.

[ref8] Chrousos G, Torpy D, Gold P (1998). Interactions between the hypothalamic-pituitary adrenal axis and the female reproductive system: clinical implications. Ann Intern Med.

[ref9] Chung F, Yao C, Wan G (2005). The associations between menstrual function and life style/working conditions among nurses in Taiwan. J Occup Health.

[ref10] Dumont C (1985). hiftwork in Asian developing countries: an overview.

[ref11] Epstein F (1997). Melatonin in humans. N Engl J Med.

[ref12] Hall J, Sullivan J, Richardson G (2005). Brief wake episodes modulate sleep-inhibited luteinizing hormone secretion in the early follicular phase. J Clin Endocrinol Metab.

[ref13] Hansen A, Garde A, Hansen J (2006). Diurnal urinary 6-sulfatoxymelatonin levels among healthy Danish nurses during work and leisure time. Chronobiol Int.

[ref14] Harlow S. D, Campbell O. M (2000). Menstrual dysfunction: a missed opportunity for improving reproductive health in developing countries. Reprod Health Matters.

[ref15] Hatch M, Figa-Talamanca I, Salerno S (1999). Work stress and menstrual patterns among American and Italian nurses. Scan J Work Environ Health.

[ref16] Jukic A, Weinberg C, Wilcox A, McConnaughey D, Hornsby P, Baird D (2008). Accuracy of reporting of menstrual cycle length. Am J Epidemiol.

[ref17] Labyak S, Lava S, Turek F, Zee P (2002). Effects of shiftwork on sleep and menstrual function in nurses. Health Care Women Int.

[ref18] Lawson C, Whelan E, Lividoti Hibert E, Spiegelman D, Schernhammer E, Rich-Edwards J (2011). Rotating shift work and menstrual cycle characteristics. Epidemiology.

[ref19] Lee J, Kang W, Yaang S, Choy N, Lee C (2009). Cohort study for the effect of chronic noise exposure on blood pressure among male workers in Busan, Korea. Am J Ind Med.

[ref20] Lisabeth L, Harlow S, Lin X, Gillespie B, Sowers M (2004). Sampling strategies for prospective studies of menstrual function. Am J Epidemiol.

[ref21] Lohstroh P. N, Chen J, Ba J, Ryan L. M, Xu X, Overstreet J. W, Lasley B. L (2003). Bone resorption is affected by follicular phase length in female rotating shift workers. Environ Health Perspect.

[ref22] Messing K, Saurel-Cubizolles M, Bourgine M, Kaminski M (1992). Menstrual cycle characteristics and work condition of workers in poultry slaughterhouses and canneries. Scan J Work Environ Health.

[ref23] Miyauchi F, Nanjo K, Otsuka K (1992). Effects of night shift on plasma concentrations of melatonin, LH, FSH and prolactin, and menstrual irregularity. Sangyo Igaku.

[ref24] Navara K, Nelson R (2007). The dark side of light at night: physiological, epidemiological, and ecological consequences. J Pineal Res.

[ref25] Pati A, Chandrawanshi A, Reinberg A (2001). Shift work: consequences and management. Curr Sci.

[ref26] Ronda E, Garcia A, Sanchez-Paya J, Moen B (2009). Menstrual disorders and subfertility in Spanish hairdressers. Eur J Obstet Gynecol Reprod Biol.

[ref27] Rowland A. S, Baird D. D, Long S, Wegienka G, Harlow S. D, Alavanja M, Sandler D. P (2002). Influence of medical conditions and lifestyle factors on the menstrual cycle. Epidemiology.

[ref28] Schernhammer E, Rosner B, Willett W, Laden F, Colditz G, Hankinson S (2004). Epidemiology of urinary melatonin in women and its relation to other hormones and night work. Cancer Epidemiol Biomarkers Prev.

[ref29] Schernhammer E, Kroenke C, Dowsett M, Folkerd E, Hankinson S (2006). Urinary 6-sulfatoxymelatonin levels and their correlations with lifestyle factors and steroid hormone levels. J Pineal Res.

[ref30] Siobán D (2000). Menstruation and Menstrual Disorders: The Epidemiology of Menstruation and Menstrual Dysfunction.

[ref31] Small C, Manatunga A, Klein M, Feigelson H, Dominguez C, McChesney R, Marcus M (2006). Menstrual cycle characteristics: associations with fertility and spontaneous abortion. Epidemiology.

[ref32] Su S, Lu C, Kao Y, Guo H (2008). Effects of 12-hour rotating shifts on menstrual cycles of photoelectronic workers in Taiwan. Chronobiol Int.

[ref33] US Bureau of the Census, Statistical abstract of the United States: the national data book (1994). US department of commerce.

[ref34] Warren M, Perlroth N (2001). The effects of intense exercise on the female reproductive system. J Endocrinol.

[ref35] Yao S. Q, Wu Q. F, Yang J. Y, Bai Y. P, Xu Y. J, Fan X. Y, Li J (2009). Effect of occupational stress on menses and sex hormones of female knitting workers. Zhonghua Lao Dong Wei Sheng Zhi Ye Bing Za Zhi.

[ref36] Zenz C, Dickerson O, Bruce O (1994). Reproductive toxicology and occupational exposure.

[ref37] Zhu J, Hjollund N, Olsen J (2004). Shift work, duration of pregnancy, and birth weight: the National Birth Cohort in Denmark. Am J Obstet Gynecol.

